# Construction and application of emergency critical care patient care plan based on Delphi method

**DOI:** 10.1097/MD.0000000000042752

**Published:** 2025-08-15

**Authors:** Hui Wang, Jing Ma, Xiaoqin Li, Meijing Yan, Panpan Zhang, Jianping Sun, Lianchun Liang

**Affiliations:** a Infection General Fever Clinic, Bejing Youan Hospital, Capital Medical University, Beijing, China; b Biomedical Information Center, Bejing Youan Hospital, Capital Medical University, Beijing, China.

**Keywords:** application effect, Delphi method, emergency critical illness, nursing plan

## Abstract

This study evaluates the efficacy of a Delphi-method-based nursing plan for critically ill emergency patients. A total of 80 critically ill patients admitted to the emergency department between January 2021 and January 2023 were included in the study, divided into 2 groups: the control group (n = 40) receiving routine emergency care, and the observation group (n = 40) receiving a nursing plan based on the Delphi method. The Delphi process involved the formation of an expert panel, followed by multiple rounds of anonymous feedback. After reviewing and refining the initial nursing criteria, the experts reached a consensus on key care indicators and interventions. The final plan emphasized personalized care, risk assessment during transport, in-hospital care, and psychological support. The observation group received care based on this plan. The transfer time, rescue success rate, rescue time, and hospitalization duration were compared, along with the incidence of complications, self-rating anxiety scale and self-rating depression scale scores, and nursing satisfaction. The transfer time, rescue time, and hospitalization duration were significantly shorter in the observation group compared to the control group (*P* < .05). The rescue success rate was significantly higher in the observation group (*P* < .05). The incidence of complications was 7.5% in the observation group, significantly lower than the 32.5% observed in the control group (*P* < .05). After nursing care, both groups showed significant reductions in self-rating anxiety scale and self-rating depression scale scores; however, the post-intervention scores in the observation group were significantly lower than those in the control group (*P* < .05). Nursing satisfaction in the observation group was 95%, significantly higher than the 80% in the control group (*P* < .05). The nursing plan for emergency critically ill patients constructed based on the Delphi method can help improve the success rate of rescue, reduce the risk of complications, enhance patients’ psychological well-being and satisfaction with care, demonstrating significant clinical implications.

## 1. Introduction

The emergency department plays a vital role in rescuing critically ill patients, and the quality of its operations directly impacts patient outcomes. Any issues arising during emergency care can pose a threat to patient safety and affect the overall efficiency of diagnosis and treatment.^[1]^ Critically ill patients often present with unclear diagnoses, unstable vital signs, and rapidly changing conditions. In-hospital transport is frequently required after initial resuscitation to facilitate further diagnosis and treatment, making it a critical component of emergency care.^[2]^ Although transport durations are typically short, serious complications such as sudden deterioration, oxygen deprivation, internal bleeding, or dislodgement of medical devices may occur, posing significant risks.^[3]^ These risks are often attributed to inadequate professional competencies of transport personnel, insufficient coordination among accompanying medical staff, rapid disease progression, and patients’ psychological stress responses. Therefore, active and effective nursing interventions are essential to reduce the incidence of adverse events.^[4]^

Traditional emergency nursing focuses primarily on executing medical orders, observing patient conditions, and administering intravenous infusions. However, it often lacks structured protocols for patient transfer and handover, which can lead to incomplete communication and omitted information, thereby endangering patient safety.^[5]^ With advancements in medical science and societal expectations, clinical demands on nurses have increased, and public expectations regarding the quality of nursing care continue to rise.^[6]^ Moreover, the majority of emergency patients are elderly, who often present with multiple comorbidities and complex conditions, further heightening the requirements for nursing care.^[7]^

The Delphi method is a structured expert consultation technique that achieves consensus through multiple rounds of anonymous feedback. It has been widely adopted in the healthcare field, particularly in clinical nursing, for developing quality standards, risk assessment tools, and care protocols – especially in areas where empirical evidence is limited.^[8–10]^ However, its application in formulating nursing care plans specifically for critically ill emergency patients remains limited and under-researched. Therefore, this study aims to develop a nursing care plan for critically ill patients using the Delphi method and to evaluate its clinical effectiveness, with the goal of enhancing patient outcomes and informing future nursing practice.

## 2. Material and methods

### 2.1. General material

A total of 80 critically ill patients who were admitted to the emergency department of our hospital from January 2021 to January 2023 were selected as the research subjects, we reviewed the case data and divided them into the control group (n = 40) and the observation group (n = 40) according to the different treatment methods. The control group was given routine emergency care, and the observation group was given the care plan for critically ill patients based on Delphi method. This study was approved by the Hospital Ethics Committee. The differences in gender, age, and disease type between the 2 groups were not statistically significant (*P* > .05). In addition, no significant differences were observed in the distribution of comorbidities (such as hypertension, diabetes, and coronary heart disease) or in baseline severity scores assessed by the rapid emergency medicine score (REMS) (*P* > .05). Therefore, the 2 groups were comparable at baseline, as shown in Table 1.

**Table 1:** Comparison of 2 groups of general material.

General material	Control group (n = 40)	Observation group (n = 40)	t/χ² value	*P* value
Gender [n (%)]			0.023	.887
Male	22 (55.00)	21 (52.50)		
Female	18 (45.00)	19 (47.50)		
Average age (years)	56.62 ± 6.87	56.56 ± 7.04	0.372	.708
Type of disease [n (%)]			0.473	.492
Pulmonary infection	36 (90.00)	38 (95.00)		
Others	4 (10.00)	2 (5.00)		
Comorbidities [n (%)]			0.105	.746
Hypertension	11 (27.50)	13 (32.50)		
Diabetes mellitus	9 (22.50)	10 (25.00)		
Coronary heart disease	7 (17.50)	6 (15.00)		
None	13 (32.50)	11 (27.50)		
Severity score (REMS)	8.21 ± 1.45	8.16 ± 1.38	0.162	.872

REMS = rapid emergency medicine score.

### 2.2. Criteria of inclusion and exclusion

Inclusion criteria: ① patients diagnosed with critical illness based on clinical evaluation and meeting the hospital’s emergency severity classification standards; ② age ≥ 18 years; ③ patients with normal cognitive function, assessed by a mini-mental state examination score ≥ 24; ④ patients who were conscious, cooperative, and able to communicate effectively; and ⑤ written informed consent was obtained from the patient or their legal guardian.

Exclusion criteria: ① patients with a confirmed diagnosis of malignant tumors; ② patients with poor clinical compliance, defined as refusal to follow medical or nursing instructions, voluntary withdrawal from treatment, or failure to complete required assessments; and ③ pregnant or lactating women.

### 2.3. Methods

Patients in the control group received routine emergency care based on the hospital’s standard operating procedures for critically ill patients. These procedures are well-established and consistently applied in clinical practice. Upon admission, patients and their families were provided with basic health education to promote cooperation. Nurses closely monitored vital signs, including heart rate, respiratory rate, blood pressure, and oxygen saturation, and promptly reported any abnormalities. Respiratory support, such as oxygen therapy and airway management, was provided as needed. Nutritional support was initiated according to clinical assessment and physician orders. For in-hospital transport, a pre-transport evaluation of patient condition and risk was conducted, and the patient’s family was informed. Nurses prepared and checked all necessary equipment, including portable ventilators and monitors, and coordinated in advance with the receiving department. During transport, patients were continuously monitored, and all lines and airways were secured. Upon arrival, a standardized handover was completed, including documentation of the patient’s condition, treatments, and any events during transfer, with confirmation by both departments.

In addition to routine emergency care, the observation group received a nursing plan for critically ill patients constructed using the Delphi method. ① Establishment of the Delphi nursing team: a multidisciplinary nursing team was formed, including the nursing department director, senior clinical nurses, and staff nurses. The team was responsible for literature review, expert recruitment, questionnaire development, and synthesis of feedback. ② Expert panel composition: 12 experts were selected based on the following criteria: (1) bachelor’s degree or higher; (2) >10 years of emergency care experience in a Class III A hospital; (3) intermediate or senior professional title; and (4) willingness to provide timely and professional feedback. ③ Delphi procedure and consensus criteria: 2 rounds of Delphi surveys were conducted. The first-round questionnaire was developed based on literature, clinical experience, and reference to the rapid acute physiology score (RAPS)^[11]^ and REMS.^[12]^ The questionnaire included expert demographics, emergency care risk factor evaluation, and self-assessment items. Experts rated each item’s importance on a 5-point Likert scale. (a) Consensus threshold: Items were retained if their mean importance score was > 3.5 and the coefficient of variation was < 0.25. (b) Modifications between rounds: Items were revised for clarity, clinical relevance, and feasibility based on round-one feedback. Items with low consensus or ambiguous descriptions were modified or removed. (c) Final item selection: Items meeting consensus criteria after round 2 were included in the final nursing care plan. ④Selection and adaptation of risk indicators: risk indicators were selected based on literature, expert consensus, and elements adapted from RAPS and REMS. These scores served as conceptual references rather than being directly applied. No formal validation was conducted in the study population. ⑤Training and protocol adherence: prior to implementation, all nursing staff in the observation group underwent structured training sessions led by senior team members. The training covered the content of the Delphi-based care plan, including procedures for first aid coordination, risk assessment, in-hospital transport, environmental care, and psychological support. Standardized protocols and checklists were distributed, and simulation scenarios were used to ensure staff proficiency. To maintain protocol adherence, a designated supervisor conducted random audits, regular team briefings, and on-site monitoring throughout the study. ⑥Bias minimization strategies: To minimize bias between groups, patients were assigned based on care plan availability rather than individual preference. Staff delivering care to each group worked independently and were blinded to the study’s objectives. The same evaluation tools and timing were used for both groups to ensure consistency in data collection. ⑦Implementation of the nursing plan: the nursing plan included multiple components: First aid coordination: establishment of an emergency nursing team, prehospital coordination, and fast-track (“green channel”) support; in-hospital transport: pre-transport risk grading, continuous monitoring, and documentation of any adverse events; environmental care: quiet, patient-centered environments with soothing elements such as light music; psychological support: Communication with patients about their condition, care plan, and emotional needs by trained personnel. Post-rescue risk registration: completion of a standardized post-event evaluation form and adverse event reporting.

### 2.4. Observational index

All outcome assessments were performed by trained nursing staff who were not involved in the intervention delivery and were blinded to group allocation. Data were collected using standardized forms and procedures to ensure consistency and reduce potential bias.

① Rescue situation: The transport time, rescue success rate (defined as: vital signs stabilized and the patient being out of critical danger), rescue time, and hospital stay were compared between the 2 groups. ② Complication incidence: the incidence of catheter-related infection, ventilator-associated pneumonia, deep venous catheter-related infection, pressure ulcer, and other complications was recorded and compared between the 2 groups. ③ Psychological status: the self-rating anxiety scale (SAS)^[13]^ and the self-rating depression scale (SDS)^[14]^ were used to assess anxiety and depression before and after nursing intervention. Both scales were self-administered by the patients under the guidance of trained staff. Each scale consists of 20 items, with a total standard score of 100 points. Higher scores indicate more severe symptoms. For the SAS, standard thresholds were applied: 50 to 59 indicates mild anxiety, 60 to 69 moderate anxiety, and ≥ 70 severe anxiety. For the SDS, scores of 53 to 62 represent mild depression, 63 to 72 moderate, and ≥ 73 severe. ④ Satisfaction with nursing: A self-developed nursing satisfaction scale was used to assess patient satisfaction in both groups. The scale was developed based on a review of relevant literature, combined with expert consultation involving 3 senior nursing professionals to ensure content validity. The scale consists of 5 items covering aspects such as communication, response time, professional attitude, nursing skills, and overall satisfaction. Each item was rated on a 3-point scale (1 = dissatisfied, 2 = relatively satisfied, and 3 = satisfied), with a total score ranging from 5 to 15. Scores of 13 to 15 were classified as “satisfied,” 10 to 12 as “relatively satisfied,” and < 10 as “dissatisfied.” The total satisfaction rate was calculated as: (number of satisfied + relatively satisfied patients) ÷ total number of patients × 100%.

### 2.5. Statistical methods

Statistical analysis was performed using SPSS 18.0. Prior to analysis, the Shapiro–Wilk test was used to assess the normality of continuous variables. For data that followed a normal distribution, results were expressed as mean ± standard deviation (x̄ ± s), and independent samples *t*-tests were used for group comparisons. For non-normally distributed data, the Mann–Whitney *U* test was applied. Categorical variables were expressed as counts and percentages (n, %) and compared using the chi-square test (χ²). A two-sided *P*-value of < .05 was considered statistically significant. No adjustments were made for multiple comparisons, as the primary outcomes were prespecified. Subgroup analyses based on disease severity (e.g., REMS scores) were not conducted due to limited sample size.

Sample size calculation: a priori power analysis was conducted using G*Power. Based on a significance level of α = 0.05, power (1 − β) = 0.8, and an estimated medium effect size (Cohen’s *d* = 0.5) from previous similar studies, the minimum required sample size was calculated to be 80 (40 per group).

## 3. Results

### 3.1. Comparison of rescue situations between 2 groups

The transport time, rescue time and hospital stay of the observation group were significantly shorter than those of the control group, and the rescue success rate was significantly higher than that of the control group. The differences were statistically significant (*P* < .05), as shown in Table 2.

**Table 2:** Comparison of rescue situations between 2 groups.

Item	Control group(n = 40)	Observation group (n = 40)	*t*/*χ*^2^ value	*P* value
Transport time (min)	41.07 ± 8.32	34.28 ± 6.98	7.645	.000
Successful rescue rate[n (%)}	8 (20.00)	1 (2.50)	6.136	.012
Rescue time (min)	49.46 ± 6.43	33.46 ± 5.69	7.301	.000
Hospital stay (d)	22.55 ± 3.45	15.52 ± 2.45	10.295	.000

### 3.2. Comparison of the incidence of complications between 2 groups

The incidence of complications in the observation group was significantly lower than that in the control group and the difference was statistically significant (*P* < .05), as shown in Table 3.

**Table 3:** Comparison of the incidence of complications between 2 groups [n (%)].

Adverse reaction	Control group (n = 40)	Observation group (n = 40)	*χ*^2^ value	*P* value
Catheter infection	3 (7.50)	1 (2.50)	–	–
Ventilator associated pneumonia	3 (7.50)	0 (0.00)	–	–
Deep venous catheter-related infection	2 (5.00)	0 (0.00)	–	–
Pressure sore	5 (12.50)	2 (5.00)	–	–
Total occurrence	13 (32.50)	3 (7.50)	7.050	.000

### 3.3. Comparison of psychological status between 2 groups before and after nursing

There was no significant difference in SAS and SDS scores between the 2 groups before nursing (*P* > .05). After nursing, the SAS and SDS scores of the 2 groups were significantly reduced, while those of the observation group were significantly lower than those of the control group, and the difference was statistically significant (*P* < .05), as shown in Table 4.

**Table 4:** Comparison of psychological status between 2 groups before and after nursing (x̄ ± s, score).

Item		Control group (n = 40)	Observation group (n = 40)
SAS score	Before nursing	54.31 ± 5.47	54.06 ± 5.74
	After nursing	49.78 ± 5.13*	41.12 ± 4.05*,**
SDS score	Before nursing	52.73 ± 6.00	52.86 ± 5.89
	After nursing	48.63 ± 5.03*	43.75 ± 4.95*,**

* *P* < .05, compared with before nursing.

** *P* < .05, compared with the control group.

### 3.4. Comparison of nursing satisfaction between 2 groups

The nursing satisfaction degree of the observation group was significantly higher than that of the control group and the difference was statistically significant (*P* < .05), as shown in Table 5.

**Table 5:** Comparison of nursing satisfaction between 2 groups [n (%)].

Satisfaction degree	Control group (n = 40)	Observation group (n = 40)	*χ*^2^ value	*P* value
Satisfied	17 (42.50)	26 (65.00)	–	–
Relatively satisfied	15 (37.50)	12 (30.00)	–	–
Dissatisfied	8 (20.00)	2 (5.00)	–	–
Total satisfaction rate	32 (80.00)	38 (95.00)	6.037	.014

## 4. Discussion

Critical patients have acute onset, serious illness, rapid disease progress, many complications and extremely high mortality, so they need to be rescued in time. The emergency department is the place for emergency patients to see a doctor and rescue critically ill patients, and it is the forefront of medical care work. Nursing is one of the important factors that affect the success of seeing a doctor and rescue, and at this time it will increase the workload of nursing staff and increase the difficulty of nursing.^[15]^ Conventional nursing is generally limited to the intervention of the disease, but can not take into account other aspects of intervention. At the same time, there are still a series of problems such as weak pertinence, incoherent time, single mode and inappropriate methods, so we need to find other more efficient nursing methods.^[16]^ Delphi method is an organization specially used for forecasting by enterprises, including a number of enterprise forecasting organizers and experts. According to the prescribed procedures, it is a method of consulting experts back-to-back for future market judgments or opinions, and then forecasting, which has the characteristics of statistics, feedback, and anonymity.^[17]^ But at present, there are few studies to explore the construction and application effect of nursing scheme for critically ill patients based on Delphi method. The results of this study show that the nursing plan for critically ill patients based on Delphi method is conducive to improving the success rate of rescue, reducing the incidence of complications, improving psychological status and nursing satisfaction. The reasons are as follows.

The condition of critically ill patients is extremely dangerous, the disease progresses rapidly, and there is a high risk of death. Clinically, in addition to active and effective treatment, nursing cooperation is also very important, while the emergency department has heavy tasks and higher requirements for nursing, and a slight mistake in rescue can lead to serious consequences, so it is very important to find an efficient nursing method.^[18,19]^ The research results of Chen et al^[20]^ show that the quality index of hemodialysis nursing sensitivity established by Delphi method can provide reliable and safe basis for the quality and safety of hemodialysis nursing in the future. The research results of Mesley et al^[21]^ show that the participants in the targeted evaluation action and monitoring clinical trials of traumatic brain injury get high satisfaction after multidisciplinary nursing. In this study, compared with the control group, the transport time, rescue time and hospitalization time of the observation group were significantly shortened, the rescue success rate and nursing satisfaction were significantly improved, and the incidence of adverse reactions was significantly reduced, which was basically consistent with the results of Chen and Mesley’s research, indicating that the nursing plan for critically ill patients based on Delphi method can effectively improve the rescue success rate and patient satisfaction, shorten the transport time, rescue time and hospitalization time, and reduce the risk of complications. The reasons are analyzed: with the improvement of people’s health needs, conventional nursing has been difficult to meet the clinical needs, and people have higher and higher requirements for clinical nursing, especially for critically ill patients, minor mistakes can pose a great threat to their life safety, so it is very important to improve their nursing quality; However, conventional nursing is lacking of pertinence and systematicness, and it is difficult to achieve clinical satisfactory nursing effect.^[22,23]^ Delphi method is a qualitative and quantitative prediction and evaluation method, which carries out anonymous expert letters, collects, sorts out and analyzes the opinions of many experts, and makes the opinions of many experts tend to be consistent after repeated letters, and then evaluates the evaluation object. At present, it has been widely used in clinical nursing work^[24,25]^; Delphi method data analysis mainly reflects the degree of expert authority, coordination coefficient, variation coefficient and positive coefficient, which can represent the recognition degree of the same problem nursing plan among peers in the professional field.^[26,27]^ The nursing plan based on Delphi method was applied to critically ill patients. According to clinical experience and referring to RAPS, REMS, and other scales, the risk factors related to first aid of critically ill patients were screened. After group discussion, the first and second rounds of expert letters were compiled, and expert letters were carried out. Experts evaluated the practicability, operability, accuracy and scientificity of each index and made suggestions. Then, important sensitive indicators for critically ill patients were constructed and corresponding nursing was implemented. Prepare the needed first-aid articles after receiving the first-aid notice, and give appropriate posture and medication guidance on the phone. When you arrive at the first aid site, do a good job in general first aid, and understand the duration of disease onset and external stimuli. After arriving at the hospital, the patient will open the green channel according to the specific situation, and then quickly carry out first aid work to improve the success rate of first aid. Do a good job in hospital transportation and environmental care. In addition, when the patient is awake and his condition is stable, he will be given psychological care to stabilize his psychology.

First of all, due to the patients’ lack of understanding of diseases and emergency, and the serious condition of critically ill patients, the intensive care unit is a special treatment place in the hospital. Affected by various instruments and equipment, such as flashing lights and noise, closed management, patients are extremely anxious and depressed, which is not conducive to the smooth implementation of emergency, so corresponding nursing intervention should be given.^[28,29]^ Borgen et al^[30]^ showed that the anxiety symptoms of patients with traumatic brain injury were obviously alleviated after 4 months of jacquard and family intervention; In this study, the scores of SAS and SDS in the 2 groups decreased after nursing, however, compared with the control group, the scores of SAS and SDS in the observation group were lower, which was basically consistent with the results of Borgen research, indicating that the nursing plan for critically ill patients based on Delphi method can effectively improve the psychological status of patients, and analyzing the reasons: the nursing plan for critically ill patients based on Delphi method has reasonable structure, systematic and comprehensive content, At the same time, the corresponding nursing measures provided by the psychological care in the scheme according to the psychological needs of patients can significantly reduce the psychological burden of patients, reduce the generation of negative emotions such as anxiety and depression, enhance self-confidence, and facilitate the smooth development of treatment.

## 5. Limitations and future directions

This study has several limitations. First, the sample size was relatively small, which may limit the generalizability of the findings and introduce potential statistical bias. Second, the study did not include long-term follow-up, making it difficult to evaluate the sustained effects of the Delphi-based nursing intervention on critically ill patients. Future research should aim to validate these findings in larger, multicenter cohorts to enhance representativeness. Additionally, incorporating long-term follow-up – such as 30-day, 90-day, or 6-month outcomes – would allow for a more comprehensive understanding of the intervention’s impact over time. Further studies could also explore subgroup analyses based on disease severity, comorbidities, or age, and investigate the cost-effectiveness and implementation feasibility of Delphi-based nursing models in different clinical settings.

## 6. Conclusion

To sum up, the nursing plan for critically ill patients based on Delphi method can not only improve the success rate of rescue, shorten the transit time, rescue time and hospitalization time, reduce the risk of complications, but also improve the psychological status and nursing satisfaction of patients, which is worthy of clinical reference.

## Author contributions

**Conceptualization:** Hui Wang, Xiaoqin Li, Meijing Yan, Jianping Sun, Lianchun Liang.

**Formal analysis:** Hui Wang.

**Funding acquisition:** Xiaoqin Li, Lianchun Liang.

**Investigation:** Hui Wang, Xiaoqin Li, Meijing Yan, Panpan Zhang, Jianping Sun, Lianchun Liang.

**Methodology:** Hui Wang, Xiaoqin Li, Meijing Yan, Jianping Sun, Jing Ma.

**Resources:** Panpan Zhang, Jing Ma.

**Supervision:** Lianchun Liang.

**Validation:** Panpan Zhang, Jianping Sun, Lianchun Liang.

**Visualization:** Panpan Zhang, Lianchun Liang.

**Writing – original draft:** Hui Wang, Jianping Sun, Lianchun Liang.

**Writing – review & editing:** Hui Wang, Jianping Sun, Lianchun Liang.
